# Plant Metabolic Gene Clusters: Evolution, Organization, and Their Applications in Synthetic Biology

**DOI:** 10.3389/fpls.2021.697318

**Published:** 2021-08-13

**Authors:** Revuru Bharadwaj, Sarma R. Kumar, Ashutosh Sharma, Ramalingam Sathishkumar

**Affiliations:** ^1^Plant Genetic Engineering Laboratory, Department of Biotechnology, Bharathiar University, Coimbatore, India; ^2^Tecnologico de Monterrey, Centre of Bioengineering, Querétaro, Mexico

**Keywords:** plant gene clusters, specialized metabolites, defensive functions, gene duplications, metabolic engineering, synthetic biology

## Abstract

Plants are a remarkable source of high-value specialized metabolites having significant physiological and ecological functions. Genes responsible for synthesizing specialized metabolites are often clustered together for a coordinated expression, which is commonly observed in bacteria and filamentous fungi. Similar to prokaryotic gene clustering, plants do have gene clusters encoding enzymes involved in the biosynthesis of specialized metabolites. More than 20 gene clusters involved in the biosynthesis of diverse metabolites have been identified across the plant kingdom. Recent studies demonstrate that gene clusters are evolved through gene duplications and neofunctionalization of primary metabolic pathway genes. Often, these clusters are tightly regulated at nucleosome level. The prevalence of gene clusters related to specialized metabolites offers an attractive possibility of an untapped source of highly useful biomolecules. Accordingly, the identification and functional characterization of novel biosynthetic pathways in plants need to be worked out. In this review, we summarize insights into the evolution of gene clusters and discuss the organization and importance of specific gene clusters in the biosynthesis of specialized metabolites. Regulatory mechanisms which operate in some of the important gene clusters have also been briefly described. Finally, we highlight the importance of gene clusters to develop future metabolic engineering or synthetic biology strategies for the heterologous production of novel metabolites.

## Introduction

Plants produce an array of specialized metabolites to evade biotic and abiotic stressors. Therefore, the production of specialized metabolites is influenced by various environmental cues. These metabolites have been extensively employed in preparing herbal formulations for human health care. For instance, specialized metabolites, such as vincristine, vinblastine, paclitaxel, and curcumin, are recognized as effective inhibitors of cell proliferation and being used in cancer therapeutics (Seca and Pinto, 2018). The significance of plant secondary metabolites in human medicine led researchers to explore the plant kingdom for understanding the biosynthetic machinery of novel metabolites. Plant specialized metabolites are classified according to their chemical backbone and functional groups. Biosynthetic pathways of several specialized metabolites have been elucidated by characterizing the pathway genes, regulators, and gene products involved in their biosynthesis. Transcriptomic, functional genomics studies combined with metabolomic approaches revealed insights into the operational features of novel metabolite pathways in different medicinal plants (Verma et al., 2014; Meena et al., 2017; Anand et al., 2019; Nagegowda and Gupta, 2020). It is quite challenging to understand the evolutionary aspects of the plant metabolic diversity at the molecular level as several metabolite-encoding gene cascades might be present in the plant genome as well, which are yet to be deciphered (Nützmann et al., 2016).

The clustering of nonhomologous genes of catabolic enzymes and the genes involved in the biosynthesis of specialized metabolites is common in prokaryotes with Lac-operon being the best example (Jacob et al., 1960). Further, in *Streptomyces* sp., genes encoding enzymes involved in the biosynthesis of antibiotics such as granaticin, actinorhodin, are reported to be clustered (Caballero et al., 1991; Ichinose et al., 1998). A few classes of filamentous fungi are known to possess clusters of both primary and secondary metabolic pathway genes and are coordinately expressed (Nützmann et al., 2018). However, the functional genes in animals and plants are scattered throughout the genome, except in a few cases of gene complexes, such as homeobox (*Hox*) and major histocompatibility complexes (*MHCs*), which exist as clusters in animals and are expressed in a synchronized manner (Horton et al., 2004; Holland, 2013; Nützmann et al., 2018). In plants, until the discovery of a gene cluster in *Zea mays* (maize) involved in the biosynthesis of hydroxamic acid 2,4-dihydroxy-7-methoxy-1,4-benzoxazin-3-one (DIMBOA), it was assumed that secondary metabolite-producing genes occur randomly in the plant genome (Frey et al., 1997). The genes encoding the enzymes for DIMBOA biosynthesis have been reported to be clustered together on the chromosome 4 in maize, and this cluster is found to be widely distributed among the monocots (Frey et al., 2009). In general, a biosynthetic gene cluster is defined as the occurrence of two or more non-homologous genes, in the vicinity on a particular chromosome, which are involved in a common biosynthetic pathway to produce a specialized metabolite or group of similar metabolites (Medema et al., 2015). In particular, plant gene cluster size ranges from 35 kb to several hundred kb, and gene clusters comprises primarily the genes responsible for determining class of metabolites and secondarily one or more genes whose role is to modulate metabolite scaffold to create metabolic diversity (Schneider et al., 2017; Nützmann et al., 2020).

Fungal and plant gene clusters generally share several similarities in the cluster architecture and evolutionary aspects except in the case of the concept of horizontal gene transfer (HGT) of clusters in fungi, which is absent in plants (Slot and Rokas, 2010; Nützmann et al., 2018). Filamentous fungi possess both primary and secondary metabolite producing gene clusters, however specialized metabolite producing gene clusters have been predominantly characterized in plants (Nützmann et al., 2018; Rokas et al., 2018). While fungal gene clusters are equipped with pathway specific transcriptional regulators and transporters, this salient feature is not common in plant gene clusters (Rokas et al., 2020). However, a few plant gene clusters have been reported to possess transporters and coordinately activated regulatory genes (Darbani et al., 2016; Hen-Avivi et al., 2016; Shen et al., 2021). In addition, multigene clusters for a single biosynthetic pathway, the co-occurrence of two clusters of genes on a single chromosome, and intertwined clusters for producing different metabolites are common in filamentous fungi (Yu et al., 2000; Bradshaw et al., 2013; Wiemann et al., 2013). These distinct features of gene cluster organization is not common in plants, except for a few cases such as the co-occurrence of steroidal glycoalkaloid- (SGA) and acyl sugar producing gene clusters on a single chromosome in the members of the *Solanaceae* and the multi-functionality of CYP76M8 in the phytoalexin production in rice (Fan et al., 2020; Kitaoka et al., 2021).

In the past two decades, more than 20 specialized metabolite-producing gene clusters involved in the biosynthesis of different classes of compounds have been identified in various plants (Nützmann et al., 2016). Recent discoveries of high-value metabolite-producing noscapine and thebaine gene clusters in *Papaver somniferum* (poppy) contributed significantly to the understanding of cluster organization, and these findings could aid in developing metabolic engineering/synthetic biology strategies for overproducing these compounds in heterologous production platforms (Guo et al., 2018). Plant metabolic gene clusters typically contain primarily the genes of committed or rate limiting enzymes of various pathways and secondarily the other enzymes required for the modification of backbone to form end-products. It has been shown that, in most cases genes are recruited from the primary metabolism through gene duplications followed by neofunctionalization (Qi et al., 2006). Most of the gene clusters are inferred to have evolved as adaptive strategies to defend against pathogen attack by producing defense metabolites, and a few other clusters are found to have a role in plant development (Qi et al., 2006; Field and Osbourn, 2008; Krokida et al., 2013; Table 1). It could be possible that plant systems favor the clustering of genes for controlled and regulated accumulation of metabolites, thus avoiding the formation of toxic intermediates and co-inheritance to progeny (Nützmann et al., 2016). Moreover, the metabolites produced by the gene clusters have a significant agronomic and human therapeutic importance, and in-depth studies are needed to develop the strategies for improving the bioproduction of target metabolites in heterologous hosts. Furthermore, plant genome mining through the genomic resources followed by functional genomic approaches together with metabolomics could reveal the existence of novel gene clusters involved in the production of high-value metabolites.

**Table 1:** Details of the gene clusters present in the different plant species and diversity of metabolites produced along with their physiological function.

**S.no**	**Species**	**Metabolite**	**Compound class**	**Chromosome (core genes)**	**Function**	**Tissue of expression**	**References**
1	*Arabidopsis thaliana*	Thalianol	Triterpenes	5	Unknown physiological function, unregulated expression of cluster genes leads to dwarfing of plant. Modulate root microbiome content	Roots	Field and Osbourn, 2008; Field et al., 2011; Chen et al., 2019a
2	*A. thaliana*	Marneral	Triterpenes	5	Unknown physiological function, unregulated expression of cluster genes leads to dwarfing of plant	Roots	Field and Osbourn, 2008; Field et al., 2011
3	*Avena strigosa*	Avenacins	Triterpenes	1	Defense against pathogens	Roots	Li et al., 2021a
4	*Cucumis sativus*	Cucurbitacins	Triterpenes	6	Insect deterrent properties and possess medicinal value	Leaves and fruits	Shang et al., 2014
5	*Ricinus communis and Jatropha curcas*	Casbenes	Diterpenes	1	Possess medicinal value and used in treating cancers and HIV infection	Constitutive expression in leaves, roots and stems	King et al., 2014, 2016
6	*Zea mays*	2,4-dihydroxy-7-methoxy-1,4-benzoxazin-3-one (DIMBOA)	Hydroxamic acids	4	Defense related activities	Mainly expressed during seedling stages and in roots.	Frey et al., 1997
7	*Oryza sativa*	Momilactones	Diterpenes	4	Insect deterring properties and anti-fungal properties	Induced expression during pathogen attack	Shimura et al., 2007; Wang et al., 2011
8	*O. sativa*	Phytocassanes Oryzalides	Diterpenes	2	Defense related activities	Induced expression during pathogen attack	Swaminathan et al., 2009; Wu et al., 2011
9	*Lotus japonicus*	Linamarin Lotuastralin	Cyanogenic glucosides	3	Herbivore deterrent activities	Above ground plant parts	Takos et al., 2011
10	*Sorghum bicolor*	Dhurrin	Cyanogenic glucosides	1	Herbivore deterrent activities	Above ground plant parts	Takos et al., 2011
11	*L. japonicus*	20-hydroxy-betulinic acid	Triterpene	3	Possible role in plant development and nodule formation	Elevated expression in roots and nodules	Krokida et al., 2013
12	*Solanum lycopersicum*	α-tomatine	Steroidal glycoalkaloid	7	Anti-pathogenic activity and toxic to humans	Elevated expression during pathogen attack	Itkin et al., 2013
13	*Solanum tuberosum*	α-solanine	Steroidal alkaloid glycoalkaloid	7	Anti-pathogenic activity and toxic to humans	Elevated during stress conditions	Itkin et al., 2013
14	*S. lycopersicum*	Mono & di terpenes	β-phellandrene Lycosantalonol	8	Involved in attracting pollinators and possible role in herbivore and fungal defenses	Localized expression in young leaves and trichomes	Matsuba et al., 2013, 2015; Zhou and Pichersky, 2020
15	*Papaver somniferum*	Noscapine	Benzylisoquinoline alkaloid	11	High value secondary metabolites	Stem and capsule	Guo et al., 2018
16	*P. somniferum*	Thebaine	Benzylisoquinoline alkaloid	11	High value secondary metabolites	Stem and capsule	Guo et al., 2018
17	*S. lycopersicum*	Falcarindiol	Fatty acid cluster	12	Elicited by pathogen attack	Above ground parts	Jeon et al., 2020
18	*Capsicum annuum*	Capsidiol	Sesquiterpene	2, 12	Pathogen induced production	Above ground parts	Lee et al., 2017

Herein, we discuss the evolutionary aspects of plant gene clusters along with their molecular features related to the organization of the clusters and regulatory mechanisms governing the organization. In addition, we elaborate the physiological role of cluster-derived metabolites in plant defense and other metabolic functions. Finally, we propose guiding principles toward the development of novel strategies related to metabolic engineering and synthetic biology by utilizing the repository of studies on plant gene clusters.

## Evolutionary Dynamics of Plant Gene Clusters

Genes that exist in close proximity on chromosomes, are often co-expressed (Elizondo et al., 2009). In prokaryotes, a set of non-homologous genes form clusters, which are generally referred as operons, and the genes in the operons are transcribed together to form a polycistronic messenger RNA (mRNA) to encode the proteins involved in a specific metabolic function (Jacob et al., 1960). The “Selfish operon model” describes that cluster arrangement of genes can improve HGT to other species, thereby increasing the chances of cluster survival (Ballouz et al., 2010). In fungi, the genes encoding enzymes involved in formation of β-lactam antibiotics and the biosynthesis of nitrate, proline, and galactose (GAL) occur as clusters with a similar pattern of co-expression (Nützmann et al., 2018). β-lactam antibiotic clusters might be of bacterial origin and could have been transferred to fungi through HGT (Liras and Martín, 2006; Slot, 2017). Similarly, GAL clusters originated differently in three species of yeast, whereas in *Saccharomyces cerevesiae* and *Candida sp*. GAL clusters are known to be originated independently through gene relocation. Comparably, in the case of *Schizosaccharomyces*, GAL cluster is found to have been acquired from *Candida* species through HGT (Slot and Rokas, 2010).

It is inferred that plant gene clusters are not evolved through HGT. Further, it has been shown that the cluster development in plants could have occurred through gene duplications, relocalization, neofunctionalization, or an independent evolution of genes toward the acquisition of specialized metabolism (Nützmann et al., 2016). To understand the evolution of plant gene clusters, Liu et al. (2020a) explored the genomes of the members of *Brassicaceae* for genome neighborhood (GNs) regions spanning around oxidosqualene cyclases (*OSCs* are key enzymes involved in sterol biosynthesis in plants) and identified that clade II *OSC*s were surrounded by cytochrome P450s (CYP450s) and acyltransferase genes possibly indicating the cluster organization. These GNs were found to be in highly dynamic chromosomal regions and lacked synteny toward the regions originated from whole genome duplication (WGD) in *Brassicaceae*, depicting an independent mode of evolution. Interestingly, functional characterization of GNs together with *OSC* in different species revealed that these putative clusters are equipped with a similar set of genes, even though they exhibited a diverse spatial and temporal expression (Liu et al., 2020a). Thalianol-producing gene cluster is known to be identical in *Arabidopsis thaliana* and *Arabidopsis lyrata*. Genome analysis of various *Arabidopsis* species revealed the evolution of thalianol cluster occurred before the divergence of *A. thaliana* and *A. lyrata*. In both the species, the cluster is organized with four core genes, whereas in *A. thaliana* three additional genes (*THAA2, THAR1 and THAR2*) are required for producing thalianin, which might have occurred through a chromosomal inversion event, but in *A. lyrata* five genes [four core genes; *THAS*, thalianol hydroxylase *(THAH), THAO, THAA1* and one linked gene; *THAA2*] are responsible for epithalianin production (Liu et al., 2020b). In addition, similar clusters from *Capsella rubella* and *Brassica rapa* are known to produce tirucallol derivatives (produced in buds) and euphol, respectively (Liu et al., 2020a). Boutanaev and Osbourn (2018) reported that transposable elements, such as miniature inverted-repeat transposable elements (MITEs), are present within the gene clusters of eudicots and they are known to play a predominant role in cluster formation by chromosomal rearrangements such as deletions, translocations, and inversions. Accordingly, triterpene clusters of *A. thaliana* are rich in transposable elements, which might have contributed to cluster formation through the segmental duplication of a committed step followed by an independent recruitment of tailoring enzymes (Field et al., 2011). In addition, transposable element-mediated genetic recombination and duplications led to the formation of sesterterpene-producing gene clusters in *A. thaliana* (Chen et al., 2019a). In addition, *Brassicaceae* members also possess pairs of terpene synthase and prenyl transferase on the genome that produces different sesterterpenes (Huang et al., 2017).

Local gene duplication could also contribute to the formation of gene clusters which are explicitly lineage specific (Schläpfer et al., 2017). For example, SGA gene clusters emerged in *Solanum lycopersicum* (tomato) and *Solanum tuberosum* (potato) through a duplication event from the common ancestor. Similar orthologous clusters have been identified in eggplant and pepper. In pepper, few genes for SGA biosynthesis underwent deletions during the course of evolution, and as a result pepper plant produces steroidal saponins instead of SGAs (Itkin et al., 2013; Barchi et al., 2019; Table 1). In addition, terpene synthase gene cluster in *Solanum* species might have evolved through several segment duplications of terpene synthase and *cis*-prenyltransferase genes, though few genes in the cluster are found to be non-functional (Matsuba et al., 2013). In *Solanaceae*, three genes *AsAT1, AACS1*, and *AECH1* are characterized to be (occuring in multi-chromosome synteny regions) responsible for the production of medium-chain acyl sugars. These genes might have evolved through the insertion and segmental duplication events that occurred in a common ancestor before divergence (Fan et al., 2020).

In monocots, avenacin cluster and metabolite biosynthesis is highly specific to *Avena strigosa* (diploid oat), and this cluster has been reported to have evolved independently (Qi et al., 2004). Genome analysis of *A. strigosa* revealed the presence of avenacin cluster in sub-telomeric regions, with the presence of the early pathway genes near to the telomere, and the existence of terminal pathway genes away from the telomere. These typical positioning co-linearity of genes in the cluster is considered to avoid the deletions of terminal pathway genes, thus preventing the accumulation of toxic intermediates (Li et al., 2021a). On the other hand, maize DIMBOA gene cluster is also located at the tip (telomeric region) of the chromosome for facilitating an adaptive evolution and a coordinated regulation (Dutartre et al., 2012). Hydroxamic acids of maize (DIMBOA) are also produced in rye and wheat, and DIMBOA-producing genes are present on two different chromosomes in respective species without disrupting metabolite biosynthesis (Frey et al., 1997, 2009). Barnyard grass, a noxious weed of rice fields, has acquired orthologous gene clusters involved in the biosynthesis of momilactone and DIMBOA, respectively. In addition, barnyard grass is known to possess a quercetin producing gene cluster, which is highly upregulated during the interaction of the weed with the host (Sultana et al., 2019). Gene clusters of rice (phytocassanes and momilactones) might have evolved as an adaptive strategy to counter pathogenic invasions (Swaminathan et al., 2009). Miyamoto et al. (2016) suggested that the evolution of these two gene clusters could have occurred in the common ancestor of *Oryza* species before domestication. In addition, momilactone cluster might have evolved through an assembly of individual genes into a physical proximity through duplications, whereas the phytocassane cluster might have already existed in the common ancestor of *Oryza* species but got lost in different lineages during the course of evolution (Miyamoto et al., 2016). Fascinating occurrence of the momilactone biosynthetic cluster in *Calohypnum plumiforme*, a bryophyte species has opened up the speculations of convergent evolution of gene clusters for species survival in challenging environments (Mao et al., 2020; Zhang and Peters, 2020). Casbene diterpenoid producing gene cluster in rice is specific to *Oryza* genus and have evolved through gene duplications (Zhan et al., 2020). Furthermore, an intact casbene production is observed in japonica cultivars (Medema et al., 2015) compared to indica cultivars of rice. This has been attributed to the natural selection of cluster in japonica varieties during the process of domestication to impart innate resistance against blight disease (Zhan et al., 2020). In addition, recent documentation of hydroxycinnamoyl tyramine producing gene cluster being specific to rice lineage and a high level of induced expression of the clustered genes during pathogen attack sheds light on the adaptive evolution of plant gene clusters in response to environmental cues (Shen et al., 2021).

In the case of cyanogenic glucoside clusters, parallel and independent evolution might have occurred in higher plants possessing the same scaffold of genes. *Lotus japonicus* and *Sorghum bicolor* are known to possess similar classes of genes in the cluster responsible for the production of plant-specific glucosides (Takos et al., 2011; Table 1). Intriguingly, an orthologous gene cluster of cyanogenic glucosides was also observed in white clover, a distant relative of *L. japonicus* (both belong to the subfamily *Papilionoideae*). They are considered to share a common ancestry, even though other members of *Papilionoideae* lost this cluster during the course of evolution as an adaptive strategy toward specific environmental niche (Olsen and Small, 2018). An *in silico* analysis using Plantismash revealed the presence of 10 orthologous gene clusters in the genomes of *Amaranthus cruentus* and *Amaranthus hypochondriacus* related to secondary metabolism. However, their *in planta* functional role is not conclusive (Ma et al., 2021). Comparative genome analysis of four genera of *Amaranthaceae* (*Amaranthus, Beta, Chenopidum*, and *Spinacia*) provided conclusive evidence about the co-occurrence of betalain pigment-producing genes on a specific chromosome, but in spinach (*Spinacia oleracea*) and quiona (*Chenopodium quinoa*) additional copies of the genes were found to exist. This could be due to tandem gene duplication mechanisms (Ma et al., 2021).

**Figure 1:**
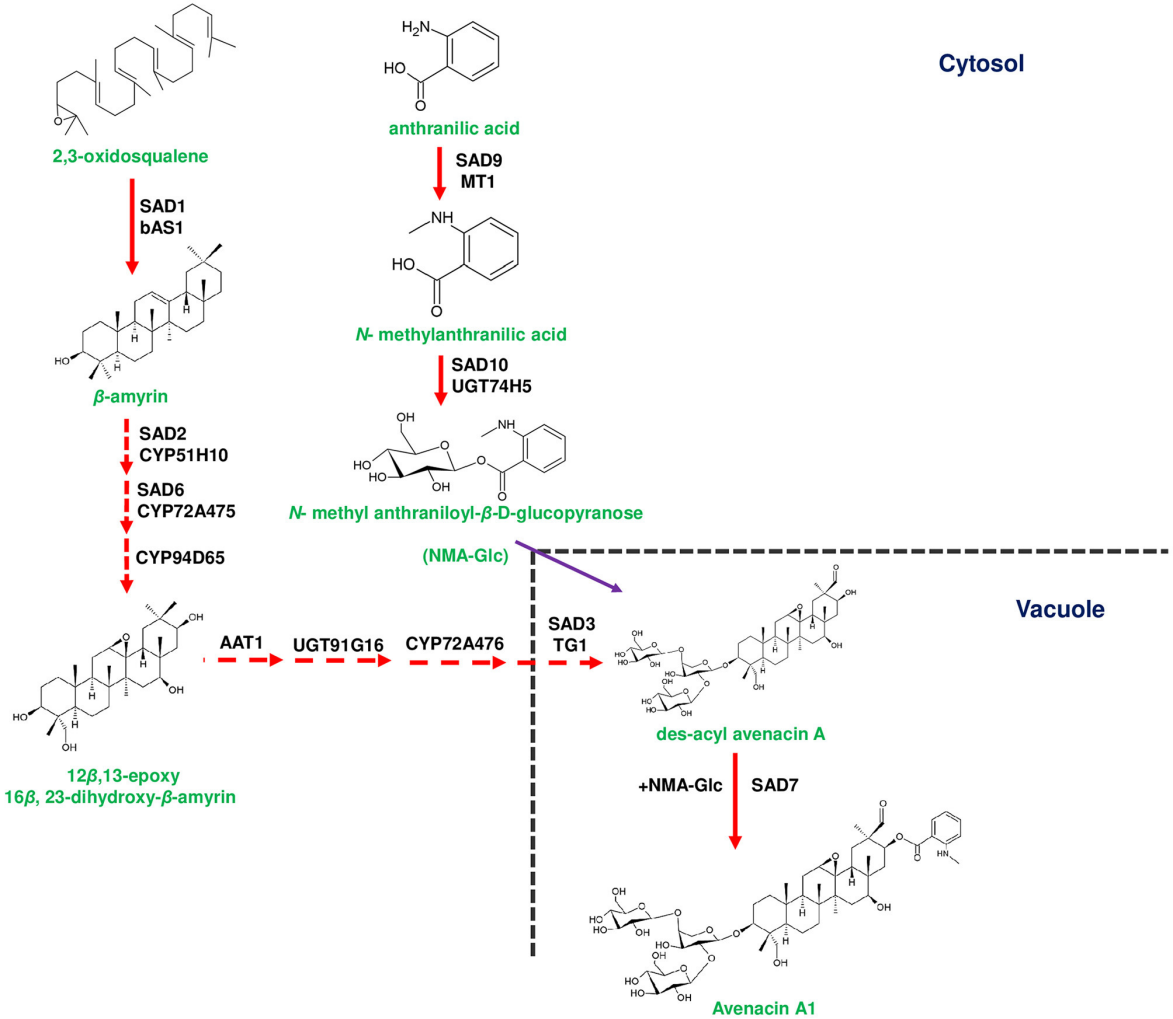
Biosynthetic pathway of avenacin in the roots of *Avena strigosa*. Avenacin biosynthesis starts with the conversion of 2,3-oxidosqaulene to β-amyrin by Sad1 (Saponin deficient 1). Conversion of β-amyrin to 12β,13-epoxy 16β, 23-dihydroxy-β-amyrin that occurs in the cytosol is mediated by Sad2, Sad6 and CYP94D65, respectively. 12β,13-epoxy 16β, 23-dihydroxy-β-amyrin is subsequently conjugated with one arabinose and two glucose moieties for the formation of des-acyl avenacin. Two glycosylation steps occur in the cytosol catalyzed by *A. strigosa* arabinosyltransferase (*As*AAT1) and *As*UGT91G16 (UDP-glucosyl transferase). The final glucose moiety is added by a unique vacuolar glycosyltransferase *A. strigosa* transglucosidase 1 (*As*TG1). Des-acyl avenacin is acylated by Sad7 aided by acyl donors such as *N*-methyl anthranilate glucopyranose (NMA-Glc) to form avenacin A1, in the vacuole. NMA-Glc is formed from anthranilic acid through a two-step reaction catalyzed by Sad9 and Sad10, respectively. Genes of the plant metabolic gene cluster encoding enzymatic reactions are indicated in red arrows. Dotted arrows represents multi-step pathway.

Available reports on gene cluster evolution indicate that plants developed gene clusters to reprogram their functional attributes toward an adaptation to different ecological niches by recruiting genes through the duplication of primary metabolism genes and acquiring new functions to them. For instance, *Sad1* (saponin deficient), *Sad2* genes in oat avenacin cluster are recruited from sterol metabolism, and novel functions were acquired subsequently (Qi et al., 2004, 2006; Figure 1; Table 2). Sonawane et al. (2016) also reported the duplication events followed by the neofunctionalization of primary metabolite biosynthesis led to the evolution of cholesterol biosynthetic genes in *Solanum* species. Moreover, the formation of these gene clusters is facilitated by various chromosomal recombination events, the presence of transposable elements, and sub-telomeric positions of clusters, and these events further support the notion that gene clusters are dynamic and evolving rapidly. Further, these gene clusters are co-inherited to progeny as an environmental adaptation for functions such as development and defense responses. In addition, negative selection pressure against the accumulation of toxic intermediates could lead to the formation of gene clusters in plants (Li et al., 2021a). Based on the observations related to the dynamic evolution of gene clusters, it is conclusive that plant genome is highly flexible and capable of associating non-homologous genes into a single coordinated cluster. Nevertheless, the lack of genome information of several plant species is a major constraint to reach a plausible conclusion on the evolution of gene clusters. It will be interesting to study the precise molecular mechanisms of evolutionary pressure that prompted plant genome plasticity. Genome mining of several plants through bioinformatics tools followed by analyzing the emergence of gene clusters in closely related and as well as distantly related species through phylogenomics, and comparative genomics approaches could help in understanding the evolutionary dynamics. Finally functional characterization of these clusters help to identify their *in planta* role as part of a major adaptive evolution.

**Table 2:** Different classes of tailoring enzymes occurring in the plant gene clusters, including CYP450s, acyl transferases, glycosyl transferases, and alcohol dehydrogenases.

**S. no**	**Cluster**	**CYP450s**	**Acyl transferases**	**Glycosyl transferases**	**Alcohol dehydrogenases**	**Other classes of enzymes**	**References**
1	Thalianol	THAH- CYP708A2 THAD-CYP705A5, THAR1, THAR2	BAHD acyltransferases THAA1, THAA2, THAA3				Field and Osbourn, 2008; Liu et al., 2020a,b; Bai et al., 2021
2	Marneral	MRO-CYP71A16 CYP705A12					Field and Osbourn, 2008; Field et al., 2011
3	Avenacin	Sad2- CYP51H10 Sad6-CYP72A475, CYP72A476 CYP94D65	Sad7 acyl transferase (Serine carboxypeptidase like protein)	Sad3–glucosyl hydrolase glycosyl transferases-Sad10, *As*AAT1 and UGT91G16		Sad9 Methyl transferase	Qi et al., 2006 and references related to avenacin biosynthesis mentioned in main text
4	Cucurbitacins	CYP81Q58, CYP89A140 CYP87D19 (within cluster) CYP87D20 (Chromosome1) CYP712D8 CYP88L2 CYP88L3 (Chromosome 3)	Acyl transferase (Csa6G088700)				Shang et al., 2014; Zhou et al., 2016
5	Casbenes	CYP76A14 CYP76A15 CYP76A16 CYP76A19 (*R. communis*) CYP71D495 CYP726A35 (*Jatropha carcus*)					King et al., 2014, 2016
6	Momilactones	CYP99A2 CYP99A3 CYP701A8 CYP76M14			*Os*MAS Short chain alcohol dehydrogenase		Shimura et al., 2007; Wang et al., 2011; De La Peña and Sattely, 2021
7	*Oryzalides* *Ph*ytocassanes	CYP71Z6 CYP71Z7 CYP76M7 CYP76M8					Swaminathan et al., 2009; Wu et al., 2011
8	Cyanogenic glucosides (*L.japonicus*)	CYP736A2 CYP79D3/D4		UGT85K2 (UDP-glucosyltransferase)			Takos et al., 2011
9	Cyanogenic glucosides (*S.bicolor*)	CYP71E1 CYP79A1		UGT85B1 (UDP-glucosyltransferase)			Takos et al., 2011
10	20-hydroxy-betulinic acid	CYP71D353					Krokida et al., 2013
11	Steroidal glucoside alkaloid cluster	GAME6 GAME7 GAME4 GAME8 (CYP72)		GAME1, GAME17, GAME18, GAME2 (UDP-glycosyl transferases)	GAME25 (Short chain dehydrogenase)	GAME11 (Putative dioxygenase) GAME12 (Transaminase)	Itkin et al., 2013; Sonawane et al., 2018
12	Falcarindiol cluster					Acetylenase Fatty acid desaturase Decarbonylase	Jeon et al., 2020
13	Capsidiol					5-epi-aristolochene synthase (EAS) 5-epi-Aristolochene 3-hydroxylase, (EAH)	Lee et al., 2017

## Organization of Plant Gene Clusters

### General Rules of Plant Gene Clusters

**Figure 2:**
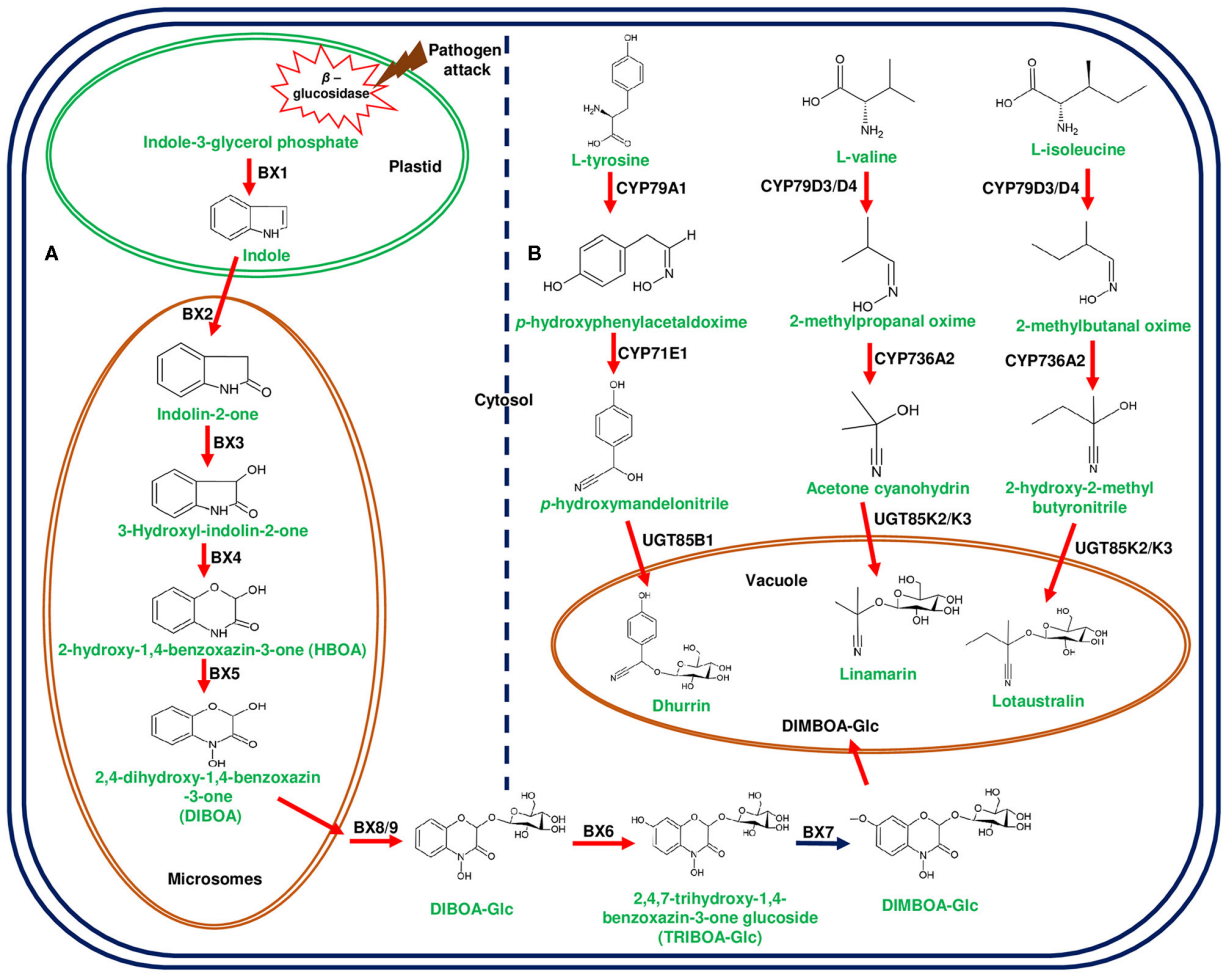
Biosynthetic pathways of 2,4-dihydroxy-7-methoxy-1,4-benzoxazin-3-one (DIMBOA) in *Zea mays* and cyanogenic glucosides in *Lotus japonicus* and *Sorghum bicolor*. **(A)** represents the biosynthesis of DIMBOA from indole-3-glycerol phosphate mediated by DIMBOA gene cluster in maize. The initial step of indole formation from indole-3-glycerol-phosphate occurs in the plastid catalyzed by benzoxazinless1 (Bx1). The conversion of indole to 2,4-dihydroxy-1, 4-benzoxazin-3-one (DIBOA) occurs in the microsomes through the catalytic reactions of cytochrome P450 enzymes (CYP450s);Bx2-Bx5. DIBOA is transported to cytosol and converted to DIMBOA-glucoside (DIMBOA-Glc) by sequential enzymatic reactions of Bx8/9, Bx6, and Bx7. The glycosylated DIMBOA is transported to the vacuole for sequestration to avoid auto-toxicity. **(B)** represents cyanogenic glucosides (linamarin, lotaustralin in *L. japonicus*, and dhurrin in *S. bicolor*) produced from different amino acids. L-tyrosine, L-valine, and L-isoleucine are converted into aglycones through the catalytic action of CYP450s belonging to CYP71 family. The final step of glucose addition is catalyzed by UGTs and sequestered in vacuole. Herbivore attack and cell disruption induces the plastidial β-glucosidase activity and releases the glucose moiety from DIMBOA-Glc and cyanogenic glucosides. Genes of the plant metabolic cluster encoding enzymatic reactions are indicated in red arrows.

**Figure 3:**
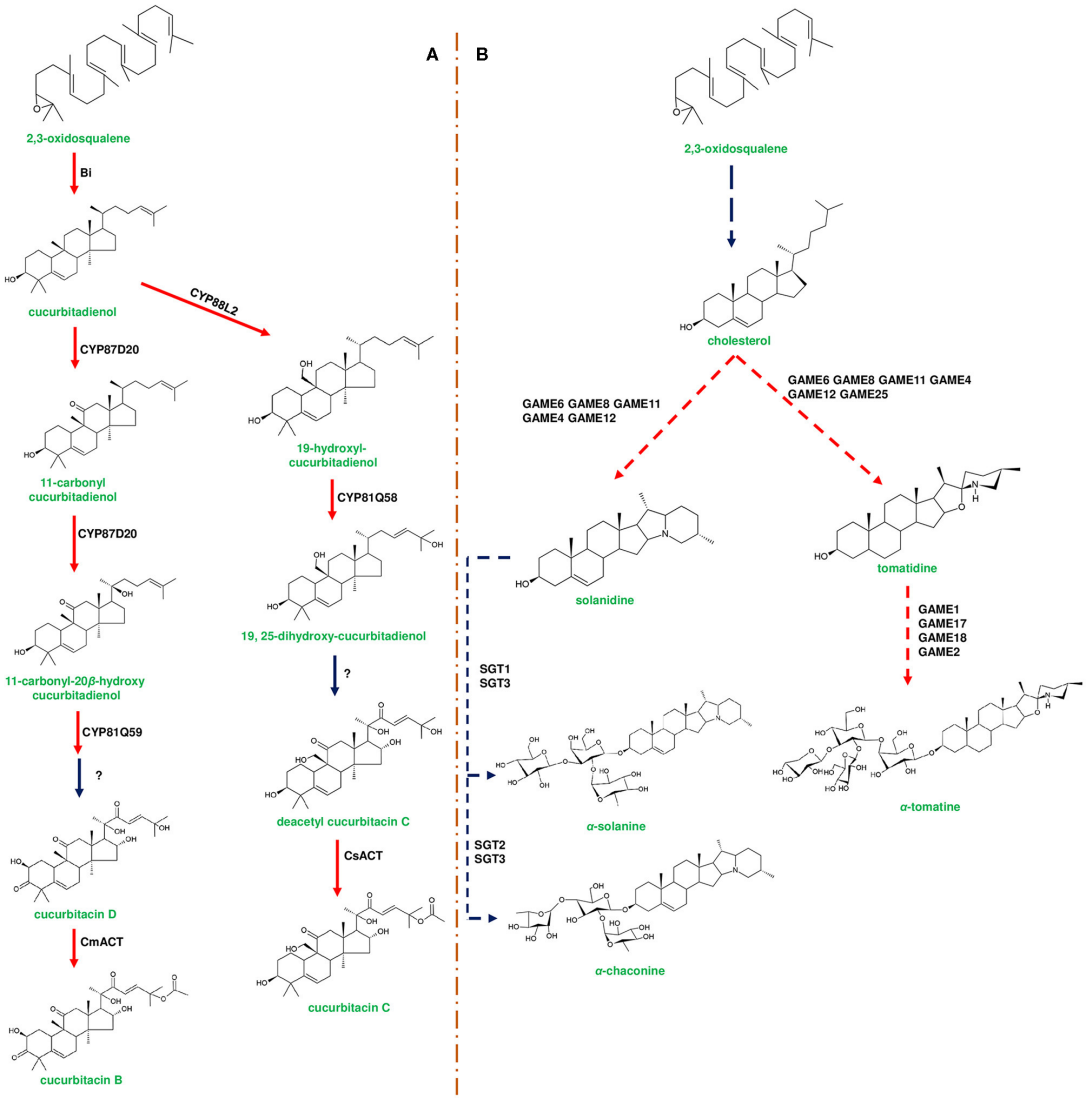
Representative biosynthetic pathway of cucurbitacins in *Cucurbitaceae* and steroidal glycoalkaloids (SGAs) in *Solanaceae*. **(A)** represents the biosynthesis of cucurbitacins through enzymes encoded by the cucurbitacin gene cluster. The first step in cucurbitacin biosynthesis occurs by the enzymatic conversion of 2,3-oxidosqualene to cucurbitadienol mediated by enzyme Bitterness (Bi) belonging to oxidosqualene cyclase family. Cucurbitadienol is converted to cucurbitacin C (CuC) in *Cucumis sativus* mediated by CYP450s and acyltransferase [*C. sativus* acyltransferase (*Cs*ACT)]. In *Cucumis melo*, cucurbitadienol is converted to cucurbitacin B (CuB) by CYP450s and *C. melo* acyl transferase (*Cm*ACT). Modification of the backbone in different *Cucumis* species is attributed to the activity of CYP450s. **(B)** represents the biosynthesis of SGAs from cholesterol using the enzymes encoded by the *SGA* gene cluster. α-solanine and α-chaconine are the signature metabolites of potato (*Solanum tuberosum*). In potato, cholesterol is converted to solanidine by catalytic reactions of GAME8 (glycoalkaloid metabolism), GAME6, GAME11, and GAME12. Further, solanidine is converted to α-solanine and α-chaconine by Sterol alkaloid glycosyl transferases (SGT1, SGT2, SGT3). α-tomatine is produced in *Solanum lycopersicum* (tomato) through a similar set of genes present in the *SGA* gene cluster. Tomatidine is formed by the modification of cholesterol mediated by GAME6, GAME8, GAME11, GAME4, GAME12, and GAME25, respectively. Tomatidine is conjugated with four sugar moieties [one moiety each of galactose and xylose and two moieties of glucose] by UDP-glycosyl transferases, GAME1, GAME17, GAME18, and GAME2, respectively. Genes of the plant metabolic cluster encoding enzymatic reactions are indicated in red arrows. Dotted arrows represent a multi-step pathway.

**Figure 4:**
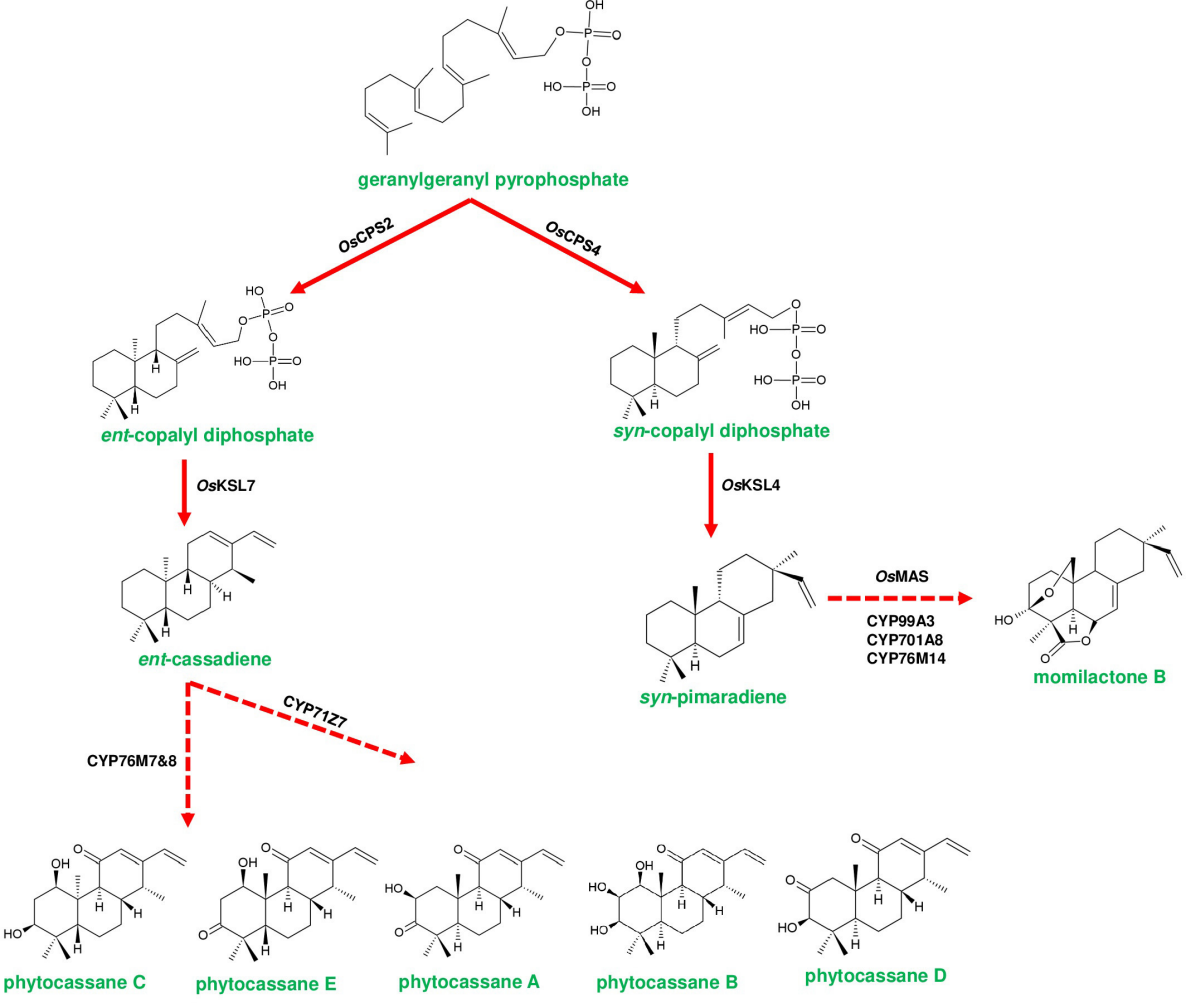
Representative biosynthetic pathway of phytoalexins in *Oryza sativa* (rice). Phytocassanes bioproduction is initiated by the conversion of geranylgeranyl pyrophosphate (GGPP) to *ent*-copalyl diphosphate (*ent*-CDP) by OsCPS2 (copalyl diphosphate synthase 2). *ent*-CDP is converted to *ent*-cassadiene by OsKSL7 (kaurene synthase like 7). *ent*-cassadiene is converted to different derivatives of phytocassanes by CYP450s. Momilactone biosynthesis is initiated by the conversion of GGPP to *syn*-copalyl diphosphate (*syn*-CDP) through OsCPS4. *syn*-CDP is converted to *syn*-pimaradiene by OsKSL4. *syn*-pimaradiene is converted to momilactone through the reactions mediated by CYP99A2/A3, CYP701A8, CYP76M14 and short-chain alcohol dehydrogenase (momilactone synthase; OsMAS). Dotted arrows represent a multi-step pathway.

**Figure 5:**
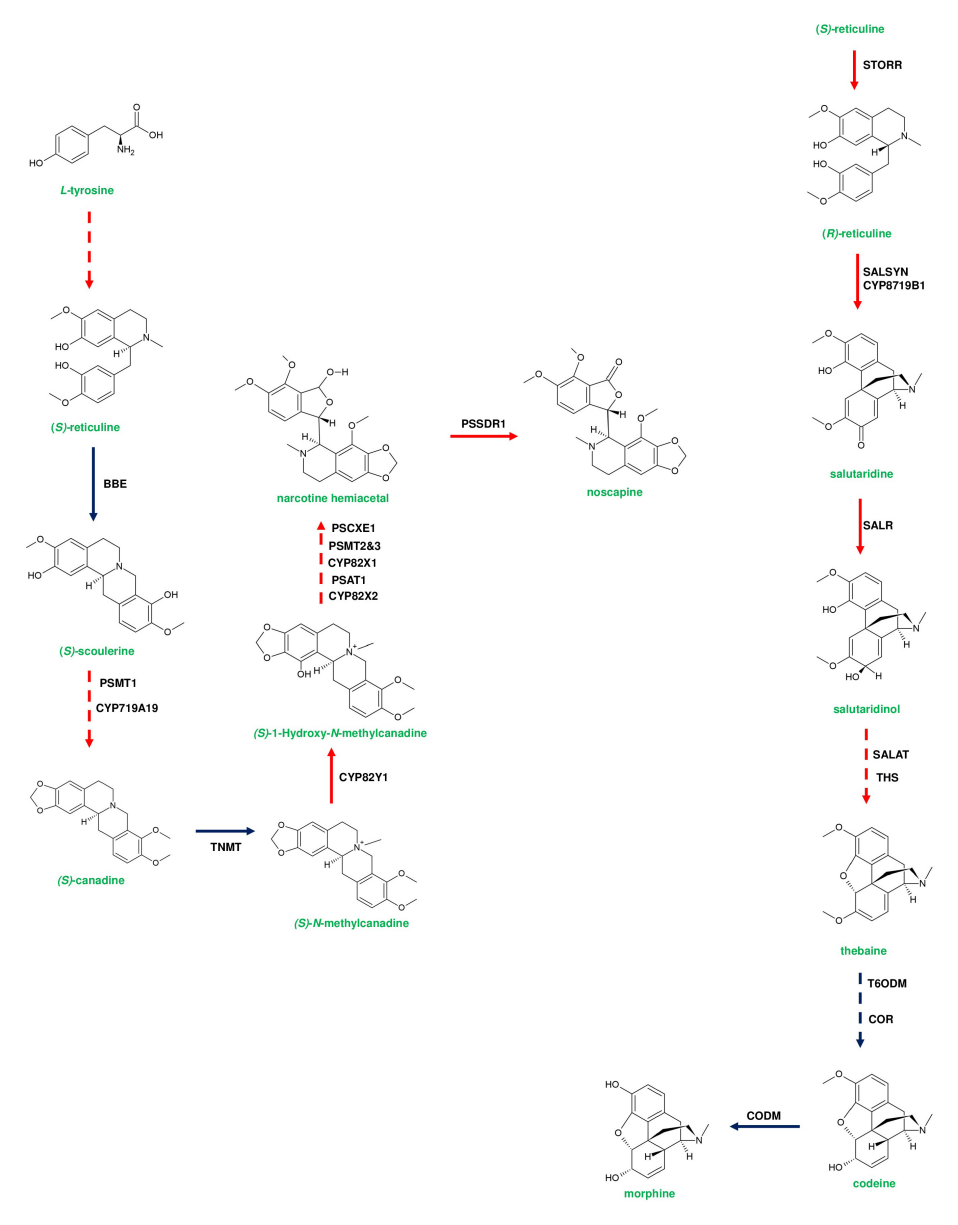
Representative pathway showing the biosynthetic events of benzylisoquinoline alkaloids (BIAs) in *Papaver somniferum*. Genes responsible for *(S)*-reticuline biosynthesis from L-tyrosine are loosely clustered in the poppy genome. *(S)*-reticuline is converted to noscapine by a set of enzymes; berberine bridge enzyme (BBE), *P. somniferum* methyltransferase 1 (PSMT1), Tetrahydroprotoberberine N-methyltransferase (TNMT), *P. somniferum* acyltransferase 1 (PSAT1), PSMT2&3, *P. somniferum* carboxylesterase 3 (PSCXE3), and *P. somniferum* shortchain dehydrogenase 1 (PSSDR1). BBE and TNMT genes are not present in the noscapine gene cluster due to their additional function in sanguinarine biosynthesis. Thebaine-producing genes are clustered and occur adjacent to the noscapine gene cluster. Thebaine biosynthesis is initiated from *(R)*-reticuline. *(S)*-reticuline is converted to *(R)*-reticuline by *(S)*- to *(R)*-reticuline (STORR) enzyme. The formation of thebaine occurs through the activity of salutaridine synthase (SALSYN), salutaridine reductase (SALR), salutaridinol-7-*O*-acetyl transferase (SALAT), and thebaine synthase (THS). Genes involved in morphine biosynthesis are not clustered. Thebaine is converted to codeine by two subsequent steps catalyzed by thebaine 6-*O*-demethylase (T6ODM), and codeinone reductase (COR). Finally, codeine is converted to morphine by Codeine3-*O*-demethylase (CODM). Genes of plant metabolic gene cluster encoding enzymatic reactions are indicated in red arrows. Dotted arrows represent a multi-step pathway.

**Figure 6:**
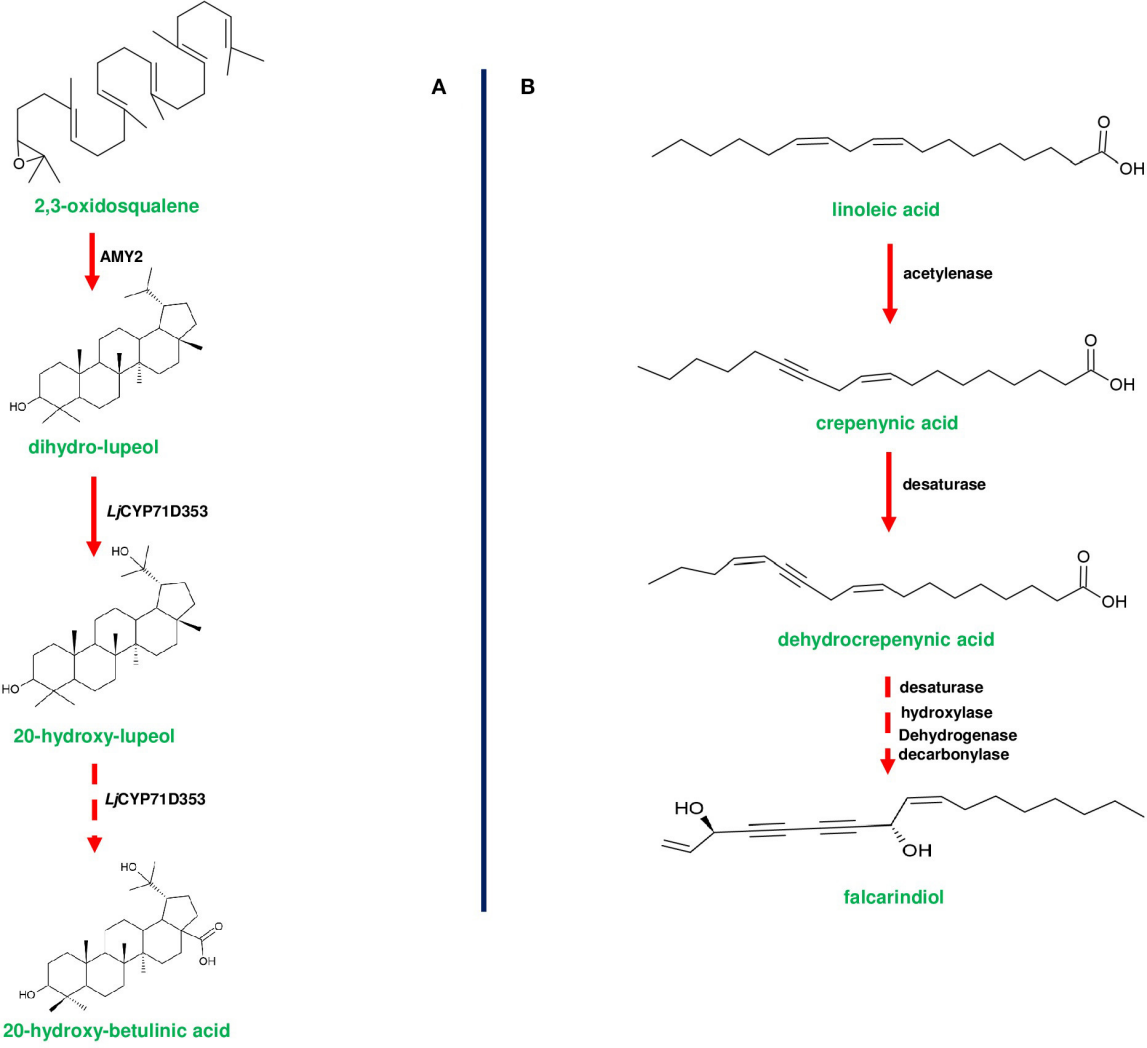
Biosynthetic pathways of 20-hydroxy-betulinic acid in *Lotus japoinucs* and anti-pathogenic fatty acid falcarindiol in tomato. **(A)** shows the biosynthesis of 20-hydroxybetulinic acid from 2,3-oxdiosqaulene by the enzymes encoded by a triterpene producing gene cluster in *Lotus japonicus*. AMY2 (β-amyrin synthase) converts 2,3-oxidosqualene into a unique metabolite dihydro-lupeol. Subsequently dihydro-lupeol is converted to 20-hydroxy-betulinic acid by two consecutive reactions catalyzed by *L. japonicus* CYP71D353. **(B)** represents the bioproduction of falcarindiol from the primary metabolite linoleic acid by the enzymes of anti-pathogenic fatty acid gene cluster in *S. lycopersicum* (tomato). Linoleic acid is converted to crepenyic acid by acetylenase enzyme encoded by *Solyc12g100240* gene. Crepenyic acid is converted into dehydrocrepenyic acid by a desaturase (Solyc12g100250). Subsequently, dehydrocrepenyic acid is converted to falcarindiol by a set of desaturase, hydroxylase, and decarboxylase. Dotted arrows represent a multi-step pathway.

Plant gene clusters have been characterized in both monocots (phytoalexin clusters in rice, DIMBOA cluster in maize, and avenacin cluster in oat) and dicots (thalianol, marneral clusters of *Arabidopsis*, noscapine, and thebaine clusters of poppy; Table 1). The common principle of gene clusters is the occurrence of set of non-homologous genes producing a specific metabolite in physical proximity on a chromosome (Medema et al., 2015; Table 1). Recent chromosomal analysis by Nützmann et al. (2020) in *Arabidopsis* revealed that the active cluster regions occur in special local hotspot regions away from the heterochromatin region and nuclear periphery. Furthermore, a similar kind of organization has been reported in rice, tomato, and maize (Nützmann et al., 2020). Genes of the first committed pathway step followed by downstream tailoring enzymes are the typical components of plant gene clusters. The number of downstream tailoring enzymes in a cluster ranges from 3 to 12 depending on the complexity of different metabolic pathways (Table 2). A signature enzyme catalyzes the first step of the pathway, which outlines the class of metabolite to be produced, and this signature enzyme is assigned to draw the primary metabolite flux toward a more specialized metabolism (Nützmann et al., 2016; Figures 1–6). However, cyanogenic glucoside, SGA pathways of *L. japonicus*, and several other members of the *Solanaceae* deviate from the above rule by catalyzing the first step of the pathway through CYP450s (Takos et al., 2011; Itkin et al., 2013; Figure 3). The arrangement of genes in plant gene clusters differs significantly, for instance, noscapine and falcarindiol clusters possess non-functional genes whose role in the metabolism is yet to be deciphered while thebaine cluster is tightly packed and contains the genes responsible for the biosynthesis of thebaine from *R*-reticuline (Guo et al., 2018; Jeon et al., 2020; Li et al., 2020; Table 1; Figure 5). In addition, genome analysis of poppy revealed that noscapine and thebaine clusters occur separately on the same chromosome while *R*-reticuline-synthesizing genes were found to be loosely clustered (Guo et al., 2018; Li et al., 2020). A similar pattern of compact clustering is also observed in oat avenacin cluster (Qi et al., 2004; Li et al., 2021a). Recently, elucidation of complete cluster organization of avenacins revealed that all genes are organized in a co-linear manner in relation to the biosynthetic steps of the pathway, and ten genes are arranged on the end of long arm of the chromosome 1 while the other two glycosyl transferases (GTs) are present in proximal scaffold (Li et al., 2021a).

Few metabolite pathway genes are known to exist adjacently in a cluster, and the three genes, *viz*., *Sad7, Sad9*, and *Sad10* are found to form an acylation module, which is responsible for acylating avenacins (Mugford et al., 2013; Nützmann et al., 2016). In contrast to the gene arrangement of avenacin cluster, *Glycoalkaloid Metabolism 7* (*GAME7*) and *GAME8* genes of SGA biosynthesis are found to be located away from the cluster in both tomato and potato (Itkin et al., 2013). *Berberine bridge enzyme* (*BBE*) and *tetrahydroprotoberberine N-methyltransferase* (*TNMT*) genes of noscapine biosynthesis are known to lack a clustered arrangement due to their additional functions in sanguinarine biosynthesis (Guo et al., 2018). In addition, two CYP450s (*THAR1* and *THAR2*) involved in the production of thalianin (the final product derived from thalianol) are located away from the thalianol cluster (Liu et al., 2020a,b). In few cases, an identical set of homologous genes are known to produce slightly different compounds. For instance, gene clusters of tomato and potato produce two different SGAs, viz., α-tomatine and α-solanine, respectively (Itkin et al., 2013; Figure 3B). Conversely, the orthologous genes of *L. japonicus* and *S. bicolor* encode a similar class of enzymes *albeit* producing different cyanogenic glucosides (Takos et al., 2011; Figure 2B). In addition, a few plants are known to possess multifunctional gene clusters equipped with the genes responsible for producing different compounds, for example, rice phytoalexin gene cluster contain the genes involved in the bioproduction of both phytocassanes and oryzalides (Swaminathan et al., 2009; Figure 4). Similarly, tomato terpene gene cluster is known to contain the genes producing mono- and di-terpenes such as lycosantalonol and β-phellandrene (Matsuba et al., 2015). In yet another situation, a few genes of monoterpene indole alkaloid (MIA) pathway are arranged together in different scaffolds in *Catharanthus roseus*. Interestingly, similar small clusters have also been observed in *Gelsemium sempervirens*, which produces the similar MIAs depicting the conserved nature of genes in different plant families (Kellner et al., 2015; Franke et al., 2019). A gene cluster producing medium-chain acyl sugars in the *Solanacaeae* occurs adjacent to the SGA gene cluster on chromosome 7. However, the evolutionary aspects regarding the co-localization of both clusters are yet to be deciphered (Fan et al., 2020). Despite the occurrence of several complicated organizational patterns of gene clusters in different plant species, the genes responsible for the bioproduction of specialized metabolites are found to be expressed in a coordinated manner independent of their occurrence within or outside the cluster. Dhurrin producing gene cluster of *S. bicolor* is found to be stringently regulated and forms metabolon for channeling intermediates, but the enzymes of avenacin and DIMBOA biosynthesis occur in different cellular compartments highlighting the complexity of cluster expression, channeling of the metabolic intermediates (Frey et al., 2009; Takos et al., 2011; Li et al., 2021a). Along with the genes responsible for metabolite bioproduction, a few clusters are also equipped with transporters and other regulatory elements. For example, the dhurrin cluster contains a co-expressed multi-antimicrobial extrusion protein (MATE) transporter gene (multidrug and toxic compound extrusion), which could bind and transport dhurrin (Darbani et al., 2016). Additionally, a cofactor synthase *OsPDX3* of rice, which is involved in the biosynthesis of hydroxycinnamoyl tyramine, occurs within the cluster (Shen et al., 2021). Hen-Avivi et al. (2016) reported the presence of a regulatory gene within the vicinity of the β-diketone cluster in wheat. Considering all the above mentioned examples, at this point of time, it is indeed difficult to draw firm conclusions related to the general organization and behavior of plant gene clusters. An in-depth analysis could reveal not only their functional role but also indicate novel evolutionary strategies of these gene clusters in conferring adaptability to the ever-changing environmental conditions.

### Significance of First Pathway Step in Metabolite Production

In most of the gene clusters identified so far, the first step of the pathway is catalyzed by a specific class of enzymes (e.g., terpene cyclases and terpene synthases) that divert the flux of primary metabolites to synthesize the cluster-specific specialized metabolites. For instance, OSC of thalianol (*THAS; thalianol synthase*) and marneral (*MRN1: marneral synthase*) clusters initiate metabolite bioproduction in *Arabidopsis* by converting 2,3-oxidosqualene, a branch point intermediate to a different set of metabolites. *THAS* and *MRN1* belong to the clade II OSC of *Arabidopsis*, and other enzymes belonging to this clade are also found to be flanked and are co-expressed (Field and Osbourn, 2008; Field et al., 2011; Liu et al., 2020a,b). Interestingly, oat-specific *A. strigosa* β*-amyrin synthase* (*AsbAS1*) gene is similar to cycloartenol synthase (has a role in sterol precursor production) and mediate the conversion of 2,3-oxidosqualene into β-amyrin (Haralampidis et al., 2001; Qi et al., 2004; Figure 1). OSC encoded by *bitterness (Bi)* gene of the cucurbitacin cluster catalyzes the first step to produce cucurbitadienol, and *Bi* is known to be highly conserved across cucurbits (Shang et al., 2014; Figure 3A). Additionally, *AMY2* (catalyzes the first step of 20-hydroxy-betulinic acid synthesis) in *L. japonicus*, encodes an unusual OSC to produce dihydro-lupeol along with a relatively lower amounts of β-amyrin (Krokida et al., 2013; Figure 6A).

Diterpene synthases of gene cluster of *Ricinus communis* are involved in the diversion of geranylgeranyl pyrophosphate (GGPP) pool from the primary metabolism to the synthesis of casbene oxidases (King et al., 2014). Similarly, phytoalexins produced by diterpene synthases of *O. sativa* (rice) share an analogy with the enzymes of gibberellin biosynthesis (Wilderman et al., 2004; Zhang and Peters, 2020). Interestingly, a tryptophan synthase homolog [*benzoxazinless1 (Bx1)*] of maize diverts the flux of indole-3-glycerol phosphate from tryptophan synthesis to indole for the biosynthesis of benzoxazinoids, and *Bx1* is considered to have evolved independently in monocots through duplication and neofunctionalization (Frey et al., 1997; Figure 2A). In addition, the identification of orthologous *Bx1* gene in dicots implicates the convergent evolution of benzoxazinoid production across the plant kingdom (Schullehner et al., 2008; Dick et al., 2012). Tomato falcarindiol cluster (a modified fatty acid) involved in the biosynthesis of specialized metabolite was shown to be initiated by acetynalase enzyme catalyzing the conversion of the primary metabolite linoleic acid to crepenyic acid (Jeon et al., 2020; Figure 6B). There are a few other gene clusters in which the first step of the metabolic pathway is not catalyzed by any signature enzyme. An example of this kind of gene cluster function is found in *L. japonicus*, where cyanogenic glucoside bioproduction is initiated by CYP450 (CYP79D3/D4), which catalyzes the conversion of amino acids (L-valine, and L-isoleucine) into respective oximes (2-methylpropanal oxime, 2-methylbutanal oxime). These oximes are subsequently converted to linamarin and lotaustralin (Takos et al., 2011; Figure 2B). CYP79 class is found to be highly conserved among higher plants and is involved in the conversion of amino acids to oximes for producing defense compounds such as glucosinolates and camalexin (Halkier et al., 2002; Takos et al., 2011). Similarly, *GAME7* encoding CYP72 class CYP450 modifies cholesterol to 22-hydroxycholestrol for SGA production in *Solanum* species (Figure 3B). Interestingly, *GAME7* was reported to be 7,880 kb away from the SGA cluster on chromosome 7 (Itkin et al., 2013; Cárdenas et al., 2016; Nützmann et al., 2016). Yet another interesting inference has been made in *Solanum* species where the biosynthetic machinery of cholesterol, the precursor for SGAs is evolved from phytosterol pathway through gene duplications and also *GAME12* of SGA pathway is likely to be evolved from gamma amino butyric acid transaminases (*GABA2*) to impart nitrogen into the steroidal backbone (Sonawane et al., 2016, 2020). In poppy, a loosely arranged gene cluster (seven genes, spanning a distance of 5 Mb across the chromosome) diverts the flux of tyrosine toward *(S)*- reticuline biosynthesis, which is the precursor for the biosynthesis of benzylisoquinoline alkaloids (BIA). On the other hand, *P. somniferum methyltransferase 1 (PSMT1)* and *(S)- to (R)-reticuline* (*STORR*) mediate the committed step in noscapine and thebaine biosynthesis, and these two genes are found to be arranged in proximity within their respective clusters (Winzer et al., 2015; Guo et al., 2018; Li et al., 2020; Figure 5).

### Tailoring Enzymes of Plant Gene Clusters: Importance of Cytochrome P450s

Plant gene clusters possess various tailoring enzymes for modifying the backbone of signature metabolite produced through the first catalytic reaction. These enzymes include CYP450s, acyl transferases, UDP-glycosyl transferases (UGTs), short-chain alcohol dehydrogenases, transaminases, and decarboxylases (Table 2). CYP450s represent the largest enzyme family playing a significant role in the structural diversification of terpene scaffolds (Ghosh, 2017). Certain classes of terpene synthases and CYP450s (TPS/CYP450) form non-random pairing and are distributed among different gene clusters in both eudicots and monocots (Boutanaev et al., 2015). Boutanaev et al. (2015) reported that TPS/CYP450s pairs might have an independent evolutionary origin among different families of angiosperms. In *Arabidopsis*, triterpene cyclases (TTC) and CYP71 clan (CYP705 family) exist as pairs in both thalianol and marneral clusters, and it has been observed that TTC/CYP71 pairs are duplicated from a single founder cluster and then both clusters independently recruited another CYP71 family genes, which include *THAH* of thailanol, marneral oxidase (*MRO*) of marneral clusters to complete the cluster organization (Field et al., 2011; Table 2). Two CYP450s of thalianol cluster are involved in the conversion of thalianol to (-)-16-keto-3β,7β,15-thaliantriol, and this metabolic intermediate is converted to thalianin upon subsequent reactions catalyzed by four gene products (two genes encoding BAHD acyl transferases: *THAA1, THAA2*, and other two oxido-reductases: *THAR1, THAR2*), a key metabolite involved in modulating microbiome of the *Arabidopsis* roots (Huang et al., 2019). Further two oxido-reductases, *THAR1, THAR2* are unlinked to the thalianol cluster and can also act on other triterpenes of *Arabidopsis* such as arabidiol and tirucalladienol resulting in their respective oxidative products (Huang et al., 2019; Liu et al., 2020a,b).

CYP71D353 belonging to the triterpene cluster of *L. japonicus* catalyzes the final steps of converting dihydro lupeol to 20-hydroxy-betulinic acid, and this enzyme is found to be phylogenetically similar to MRO (CYP71A16; marneral oxidase) of marneral cluster belonging to CYP71 clan CYP450s (Krokida et al., 2013; Figure 6A). Additionally, it is reported that CYP71 clan enzymes can oxidize various triterpenes, such as, lupeol, β-amyrin, and α-amyrin, leading to more specialized metabolites (Yasumoto et al., 2016). TTC/CYP81Q58 pair along with CYP88L2 and *Cs sativus* acyl transferase (*cs*ACT) accounts for the bioproduction of cucurbitadienol and its further conversion to cucurbitacin C (CuC) in *Cucumis sativus* (Shang et al., 2014). Interestingly, the other two *Cucumis* species (musk melon and water melon) do not produce CuC due to non-functional CYP88L2, instead they produce cucurbitacin B (CuB) and cucurbitacin E (CuE) through the catalytic activity of CYP87D20 and CYP81Q59, respectively (Zhou et al., 2016; Figure 3A). Moreover, CYP450s and tissue specific regulators involved in the biosynthesis of cucurbitacins were differentially expressed in both wild and domesticated species of *Cucumis*, indicating the operation of environmental selection force on cluster organization (Zhou et al., 2016).

Rice phytoalexin gene cluster (phytocassanes and oryzalides) has a combination of diterpene synthase genes with CYP450s of CPY71 clan (TPS/CYP71). GGPP is converted into *ent*-copalyl diphosphate (*ent*-CDP) by CPS2, then two kauerene synthase like genes (*KSL6* and *KSL7*) produce *ent*-isokaurene and *ent*-cassadiene from *ent*-CDP, which are the immediate precursors for oryzalides and phytocassanes, respectively (Wu et al., 2011; Figure 4). CYP71Z6 mediates primarily the biosynthesis of oryzalides, and on the other hand, CYP76M7 & CYP76M8 and CYP71Z7 catalyze subsequent steps of phytocassane biosynthesis (Swaminathan et al., 2009; Wu et al., 2011; Figure 4). Similarly, rice momilactone cluster contains CDP synthase (*OsCPS4*), kaurene synthase like (*KSL4*), CYP99 family (*CYP99A2/CYP99A3*) genes, and dehydrogenase (*Os*MAS), which are collectively involved in the production of momilactone (Shimura et al., 2007; Wang et al., 2011; Figure 4). The elucidation of the momilactone pathway by De La Peña and Sattely (2021) led to the identification of two other CYP450s (CYP701A8 and CYP76M14) that are responsible for the final conversion of momilactone A to momilactone B (De La Peña and Sattely, 2021). Furthermore, Kitaoka et al. (2021) proposed a novel biosynthetic route of momilactone biosynthesis through deciphering the role of CYP76M8, which converts *syn*-pimaradien-19-al to 6β-hydroxy-*syn*-pimaradien-19-al, a key intermediate in momilactone production. Afterward, this metabolite gets converted to momilactone through subsequent steps catalyzed by *Os*MAS1 or *Os*MAS2 and CYP701A8, respectively (Kitaoka et al., 2021). CYP76M8 is located in the phytocassane cluster, which is a close relative of CYP76M7. However, the functioning of CYP76M8 together with momilactone cluster genes for metabolite biosynthesis indicates the interdependent evolution of gene clusters as a selective advantage (Kitaoka et al., 2021). The promiscuous activity of CYP76M8 is further supported by its ability to hydroxylate several diterpene metabolites of rice (Wang et al., 2012). Moreover, the bryophyte *C. plumiforme* possess four genes *CpDTC1*/*HpDTC1, CpMAS, CpCYP970A14*, and *CpCYP964A1* related to momilactone production, and the complete biosynthetic pathway is yet to be elucidated (Mao et al., 2020; Zhang and Peters, 2020).

CYP726A14 and CYP726A15 of casbene cluster in *R. communis* perform the conserved step reaction by oxidizing casbene and neocembrene at the fifth position, and the oxidation of the fifth carbon atom is conserved among the casbene-derived metabolites distributed across *Euphorbiaceae* (King et al., 2014). In *Jatropha curcas*, CYP726A35, orthologous to the CYP726A18 oxidizes the fifth position of casbene metabolites (King et al., 2016). Few other CYP450s (CYP726A19, CYP71D495) in the clusters of *R. communis* and *J. curcas* catalyzes the oxidation of casbene and its derivatives for further metabolic diversification (King et al., 2014, 2016). Recently, Zhan et al. (2020) identified a similar 5,10-diketo-casbene producing gene cluster (*OsTPS28, OsCYP71Z2, and OsCYP71Z21*) on the seventh chromosome of rice while *Os*TPS28 converts GGPP to casbene and the other two CYP450s converts casbene to 5,10-diketo-casbene (Zhan et al., 2020).

Oat avenacin cluster possesses an unique combination of TTC and CYP51 (*AsCyp51H10*) encoding enzymes of the first two steps of avenacin biosynthesis (Figure 1). CYP51 family has been shown to play a specific role in sterol biosynthesis, but it has been demonstrated that *AsCyp51H10* was recruited from ancient CYP51 family and acquired new functions in avenacin metabolism (Haralampidis et al., 2001; Qi et al., 2006; Figure 1). On the other hand, CYP51 gene of sterol biosynthesis in *Solanum* species performs additional function in cholesterol biosynthesis, which is a precursor of SGAs (Sonawane et al., 2016). Moreover, oat avenacin cluster has been proposed to have evolved independently and orthologous genes and metabolite production were not found in other monocots (Qi et al., 2004). Meanwhile, similar pairing of TTC/CYP51 is observed in a cereal member, *viz*., *Brachypodium distachyon*, its functional similarity with the avenacin cluster is still found to be inconclusive (Boutanaev et al., 2015). Along with *CYP51H10*, three more CYP450s have been shown to be associated with avenacin biosynthesis, out of which two CYP450s belong to the CYP72 clan. CYP72A475 and CYP72A476 participate in C-21-β-hydroxylation and C-30 aldehyde group addition to β-amyrin scaffold, respectively (Leveau et al., 2019; Li et al., 2021a). CYP94D65 is responsible for adding a hydroxyl group at C-23 position of β-amyrin scaffold (Li et al., 2021a). In a comparable way, CYP71C family genes (*Bx2* to *Bx5*) of maize involved in DIMBOA biosynthesis are of monophyletic origin and homologous genes were not observed in other monocots other than *Bx2* (Frey et al., 2009; Figure 2A). These set of genes might have evolved before divergence of *Triticeae* and *Panicoideae* (Frey et al., 2009).

The non-random pairing of TPS and CYP450s were also present in the genomes of the members of *Lamiaceae* (Lichman et al., 2020). For instance, the genome assembly of *Tectona grandis* revealed the presence of 41 terpene synthase genes in 14 tandem clusters, and 20 TPS together with 31 CYP450s were physically clustered in the genome. *TPS-c*/*CYP76AH31* combination of *T. grandis* is involved in diterpene biosynthesis displaying a high percentage of homology toward *Salvia miltiorrhiza Sm*CPS1/CYP76AH12 responsible for the production of tanshinones (Zhao et al., 2019). In the case of *S. miltiorrhiza SmCPS1* and *SmCPS2*, genes were flanked with CYP76AH subfamily genes involved in tanshinone biosynthesis, whereas these clusters might have formed from the ancestral duplication event of *CPS*/*CYP76AH* pair (Xu et al., 2016). Similarly, in *Salvia splendens* genome, eight clusters of TPS/CYP450 combinations were found, even though the functional role of these combinations are yet to be deciphered (Dong et al., 2018). The occurrence of gene pairs of terpenoid biosynthesis is not restricted to TPS and CYP450 combinations alone in *Brassicaceae*. The distinct pairs of prenyl transferase and terpene synthases were spread across the genome of several members, which are responsible for the biosynthesis of sesterterpenoids and these pairs might have evolved from a common ancestral pair *via* duplication and functional divergence (Huang et al., 2017). It has been reported that the construction of Lavender (*Lavandula angustifolia*) genome assembly led to the identification of TPS-TPS, TPS-BAHD acyltransferases, and TPS-CYP450 gene pairs, which are found to be induced upon stress conditions (Li et al., 2021b). It has been inferred that tandem duplications might have been the driving force for the emergence of these gene combinations (Li et al., 2021b). In addition, it has been reported that the coupling of terpene synthases and CYP450s P450s in gene clusters are conserved in eudicots, but not in monocots. The occurrence of transposable elements (in eudicots) and the sub-telomeric position of clusters (in monocots) indicate the possibilities of the occurrence of recombination events leading to a novel cluster formation resulting in metabolic diversity (Qi et al., 2006; Field et al., 2011; Boutanaev et al., 2015).

### Acyl Transferases and Glycosyl Transferases: Avoiding the Accumulation of Toxic Intermediates

Plants have evolved in such a way to physically link genes as clusters to avoid the accumulation of toxic intermediates (Nützmann et al., 2016). The addition of sugar moieties and acyl groups to metabolic pathway intermediates, respectively, through glycosyl transferases and acyl transferases results in reducing the cytotoxicity (Itkin et al., 2011). It is known that the biological activity of triterpenes requires acylation at C-21 position (Podolak et al., 2010). Accordingly, des-acyl avenacins are acylated at C-21 position by serine carboxypeptidase like (SCPL) acyltransferase (Sad7), which incorporates *N*-methyl anthraniloyl group to produce avenacin A1 (Mugford et al., 2009; Table 2; Figure 1). In general, SCPL acyltransferases utilize β-acetyl glucose esters as acyl donors and, in oat Sad7 utilizes *N*-methyl anthraniloyl-*O*-glucopyranose (NMA-Glc) as the acyl donor (Mugford and Osbourn, 2010; Ciarkowska et al., 2019). NMA-Glc is synthesized from anthranillic acid through two sequential steps catalyzed by methyltransferase (Sad9) and UDP-glucosyltransferase (Sad10), respectively (Owatworakit et al., 2012; Mugford et al., 2013; Figure 1). It is interesting to note that *Sad7, Sad9*, and *Sad10* occur adjacently in the avenacin gene cluster and regarded as acylation module (Mugford et al., 2013). In addition, Sad7 can acylate des-acyl avenacins with benzoyl group donated by benzoyl-β-glucopyranose to form avenacin A2 (Mugford et al., 2009; Owatworakit et al., 2012). Hence, the scaffold diversity of avenacins is attributed to the SCPL acyltransferase (Sad7) and availability of acyl donors (Figure 1).

Besides acylation, triterpene backbone of avenacin is glycosylated with branched trisaccharide moiety (one arabinose and two glucose molecules), which is essential for its antimicrobial activity (Louveau et al., 2018). In the first instance, the addition of arabinose to β-amyrin scaffold is mediated by an arabinosyltransferase (*As*AAT1) and the mutants of *asaat1* exhibited susceptibility to pathogenic *Gaeumannomyces* sp. (Louveau et al., 2018). Subsequently, two glucose molecules are attached to L-arabinose through glucosyltransferase and glycosyl hydrolase encoded by *AsUGT91G16* and *A. strigosa* transglucosidase 1 (*As*TG1), respectively (Figure 1). *As*UGT91G16 adds 1,2 linked glucose to arabinose, and *As*TG1 performs the final addition of 1,4 linked glucose (Orme et al., 2019). Remarkably, *AsTG1* (synonymous to *Sad3*; a core gene of avenacin cluster) encodes a unique vacuolar glycosyl hydrolase 1 (GH1), and the final addition of glucose moiety is known to occur in the vacuole, although an activity of glycosyl transferases is predominant in the cytosol as well (Hansen et al., 2013; Orme et al., 2019; Figure 1). Furthermore, *sad3* mutants accumulated non-glycosylated avenacins, which resulted in stunted root growth and deformed root hairs in oat, implicating the importance of glycosylation for normal avenacin bioactivity (Mylona et al., 2008). While the core cluster of avenacin exists in a sub-telomeric region of chromosome 1, *AsUGT91G16* and *AsTG1* genes exist in a location away from the core cluster in a proximal scaffold to avoid telomere gene deletions, which in turn help to avoid the accumulation of toxic intermediates (Li et al., 2021a). In *Arabidopsis*, three BAHD acyltransferases, *THAA1, THAA2*, and *THAA3*, are involved in triterpene metabolism, and acetylates signature metabolites, such as thalianol, arabidiol, and deletions of the *THAA2* locus, could lead to the development of short roots as compared to the wild type although an internal molecular cue is yet to be deciphered (Huang et al., 2019; Liu et al., 2020a,b; Bai et al., 2021).

Plants producing benzoxazinoids (DIMBOA) glycosylate them to avoid auto toxicity and store these biomolecules in the vacuole, while during defense response β-glucosidase cleaves sugar molecule and activates the function (Von Rad et al., 2001; Dick et al., 2012; Figure 2A). *Solanum* species also glycosylate SGAs to avoid auto toxic effects, and four UGTs (*GAME1, GAME17, GAME18*, and *GAME2*) of α-tomatine gene cluster adds tetrasaccharide moieties (one galactose, one xylose, and two glucose molecules) to tomatidine for the formation of α-tomatine (Itkin et al., 2013; Figure 3B). Similarly, α-solanine and α-chaconine are glycosylated with glucose:galactose:rhamnose and rhamnose:galactose:rhamnose, respectively (Ohyama et al., 2013). Alterations in GAME1 activity have been shown to result in the accumulation of high levels of tomatidine and as a consequence plants exhibited defective fruit development and increased susceptibility to pathogen attack. It has been reported that the accumulation of α-tomatine and its subsequent conversion to esculeoside A is important for the normal development of tomato fruits (Itkin et al., 2011). Additionally, *GAME25* encodes a short-chain dehydrogenase, which catalyzes the reduction of a double bond at the C-5,C-6 position and is considered as a key step in determining the diversity of SGAs in *Solanum* species. In addition, this modification has been shown to result in the reduction of the toxic effects of SGAs (Sonawane et al., 2018).

In a similar way, cyanogenic glucosides are glycosylated at the final step and transported to the vacuole (Gleadow and Møller, 2014). Cyanogenic glucoside producing the gene clusters of *L. japonicus* and *S. bicolor* are equipped with UGTs so as to catalyze the final glycosylation step for producing linamarin, lotaustralin, and dhurrin (Takos et al., 2011; Figure 2B). Independent recruitment of genes and their coordinated expression for metabolic channeling are the characteristic features of plant gene clusters to avoid the accumulation of toxic metabolic intermediates. Extensive mining of plant genomes can reveal the novel gene clusters, and it would be interesting to study their architecture related to the arrangement of the constituent genes of the cluster and their functional attributes in metabolite production.

## Regulation of Plant Gene Clusters

### Plant Gene Clusters Are Stress-Induced and Exhibit Spatiotemporal Expression

In general, plant gene clusters are spatiotemporally regulated (Qi et al., 2006; Shimura et al., 2007; Table 1). The expression of *AsbAs1* of avenacin cluster and other genes are found to be localized specifically in root tips, depicting that the biosynthesis and storage of metabolites are tissue specific (Haralampidis et al., 2001; Qi et al., 2004, 2006). In maize, the biosynthesis of DIMBOA is reported to be developmentally regulated with a localized expression restricted to leaves and roots (Frey et al., 1997). The expression of thalianol and marneral gene clusters of *Arabidopsis* are localized in root epidermis. However, the physiological function of the clusters is yet to be deciphered. The overexpression of these clusters resulted in dwarfing and negatively affected the overall plant development in *Arabidopsis* (Field and Osbourn, 2008; Field et al., 2011). In addition, triterpenes produced by *Arabidopsis* roots are reported to modulate the microbial diversity in the rhizosphere by promoting the enrichment of species-specific bacteria on root surfaces (Huang et al., 2019) though the role of microbiota in plant development is not conclusive. Mutant *Arabidopsis* lines of triterpene biosynthetic genes significantly reduced microbial operational taxonomical units (OTUs) compared to wild type (Huang et al., 2019). In continuation with this, Chen et al. (2019a) identified a group of sesterterpene cluster-derived metabolites in *A. thaliana*, which were shown to modulate the density of root microbiota. This observation was further confirmed by analyzing OTUs in wild-type and mutant lines (Chen et al., 2019a).

Diterpene clusters in rice, such as phytocassanes, momilactones, and oryzalides, are activated by chitin oligosaccharide-based elicitation and UV irradiations, and their biosynthesis is localized in leaves and roots (Shimura et al., 2007; Swaminathan et al., 2009). Pathogen-induced capsidiol gene cluster has been identified in plants such as capsicum and tobacco, and the cluster is highly conserved in both the species (Lee et al., 2017; Chen et al., 2019b). CYP450s catalyzing the biosynthesis of linamarin and lotaustralin was found to be highly expressed in younger apical leaves and the expression gradually diminished with plant age (Takos et al., 2011). In a similar way, the expression of lycosantalonol-producing genes leaf petiole, and β-phellandrene gene expression is confined to leaves (Matsuba et al., 2015; Zhou and Pichersky, 2020). Interestingly, 20-hydroxy-betulinic acid-producing gene cluster in *L. japonicus* is highly expressed during nodule formation, thus portraying its role in plant development (Krokida et al., 2013). Therefore, it is evident that gene clusters evolved as adaptive strategies to produce defense-specific metabolites in a rapid and coordinated fashion to evade both biotic and abiotic stress conditions.

### Role of Transcription Factors and Chromatin Remodeling

Three basic helix–loop–helix (bHLH) transcription factors (TFs) related to cucurbitacin biosynthesis have been identified in cucurbits, *viz*., *bitter fruit* (*Bt*), *bitter leaf* (*Bl*), and *bitter root* (*Br*), and the expression of these TFs is specific to fruits, leaves, and roots, respectively. These regulators can strongly bind to the promoter regions of nine cucurbitacin biosynthesis genes and influence the biosynthesis of CuC, CuB, and CuE (Shang et al., 2014; Zhou et al., 2016). In addition, it has been shown that domesticated cucurbit fruits (cucumber, melon, and watermelon) lost their bitterness due to a mutation in the *Bt* gene affecting its binding properties to promoter regions, although the wild species retained Bt activity (Zhou et al., 2016; Chomicki et al., 2020). In tomato and potato, *GAME9* TF [belonging to a class of Ethylene Responsive Factor (ERF)] is found to regulate SGA bioproduction. While the overexpression of *GAME9* leads to increased levels of α-tomatine, α-chaconine, the knockout of *GAME9* activity reversed the expression of SGA biosynthetic genes (Cárdenas et al., 2016). Recently, Yu et al. (2020) identified two allelic variants (GAME9^135A^ and GAME9^135V^) of GAME9 in wild and domesticated *Solanum* species. In the wild species, GAME9^135A^ exhibited a strong binding affinity to *GAME7* and *GAME17* gene promoters (Yu et al., 2020). However, GAME9^135V^ did not display strong interactions with the promoter regions and is coordinated with another TF MYC2 to regulate SGA biosynthesis (Yu et al., 2020). In addition, JRE4 TF of tomato increased SGA accumulation by binding to the promoter region of *GAME4* gene. In addition, overexpression and knockout experiments established the role of JRE4 in SGA biosynthesis (Thagun et al., 2016; Nakayasu et al., 2017).

In rice, elicitor-induced bZIP TF *OsTGAP1* has been identified along with its possible role in momilactone biosynthesis, and it has been shown that the overexpression of *OsTGAP1* significantly enhanced metabolite accumulation (Okada et al., 2009). Intriguingly, *Os*TGAP1 does not directly regulate the transcription of the momilactone cluster, instead it binds to the intergenic regions adjacent to the momilactone cluster (Miyamoto et al., 2014). However, *Os*TGAP1 binds strongly to the promoter region of *OsDXS* gene and upregulates its transcription, probably to increase the precursor pool (Miyamoto et al., 2014). Interestingly, the overexpression of TGA factor, *Os*bZIP79 suppresses momilactone biosynthesis, and *Os*bZIP79 and *Os*TGAP1 interact with each other to form a heterodimer, which might have a role in phytoalexin biosynthesis in rice (Miyamoto et al., 2015). In addition, TFs regulating the synthesis of nicotine and terpene indole alkaloids are clustered in the genome for facilitating a coordinated activity in *Nicotiana tabacum* and *C. roseus*, respectively (Shoji et al., 2010; Singh et al., 2020).

In *Arabidopsis*, metabolic clusters possess histone 3 lysine trimethylation (H3K27me3) and histone 2 variant H2A.Z chromatin signatures regulated by sick with RSC/Rat1 (SWR) complex-mediated chromatin remodeling (Yu et al., 2016). Actin-related protein (Arp6), a subunit of SWR complex, incorporates a histone 2 variant (H2A.Z) into nucleosomes of cluster genes and facilitate metabolite bioproduction. The downregulation of the cluster gene expression in the mutants of *arp6* and *h2a.z* further confirmed their regulatory role in the cluster (Nützmann and Osbourn, 2015). Reimegård et al. (2017) identified the similar modifications performed by histone methylases and histone deacetylases to facilitate the expression of development-specific gene clusters of *Arabidopsis*. Zhan et al. (2020) reported a histone demethylase JMJ705 activated upon methyl jasmonate treatment, which acted antagonistically on H3K27me3 of a chromatin region and upregulated the expression of rice casbene gene cluster. Recently, Nützmann et al. (2020) reported that metabolic gene clusters occurred in the local interactive domains of plant genome, and these domains surrounding the clusters facilitated the tightly coordinated expression of genes in the cluster. Further, the localization of the silenced clusters occurred in the periphery of the chromosomes while the expressing clusters are positioned in a location away from the periphery (Nützmann et al., 2020). In addition, chromatin-level remodeling is evident in the regulation of specialized metabolite gene clusters in filamentous fungi (Bok et al., 2009). Wegel et al. (2009) demonstrated that chromatin decondensation at *Sad1, Sad2* genes occurs only in root tip epidermis to initiate avenacin synthesis. Similar decondensation process was minimal in other tissues, although internal molecular cascades of cell-specific chromatin decondensation is yet to be understood.

## Physiological Activities of Plant Gene Cluster-Derived Metabolites

Metabolites produced by gene clusters exhibit a specific role in defense responses and few other metabolites are involved in regulating the cell physiology during plant development (Krokida et al., 2013; Louveau et al., 2018; Table 1). Avenacins synthesized by oat exhibit antifungal properties and are known to inhibit fungal infection, by acting against *Blumeria graminis, Bipolaris oryzae*, and *Magnaporthe oryzae* (Inagaki et al., 2013). Non-glycosylated avenacins (*Sad3* mutants) severely affected the formation of root epidermis, implicating the biosynthesis and accumulation of specific intermediates of specialized metabolites is cytotoxic to the plant (Mylona et al., 2008). On the other hand, rice momilactones are induced by UV irradiation and are active against a wide range of fungal pathogens such as *Magnaporthe grisea, Botrytis cinerea, Fusarium solani, Colletrotrichum gloeosporioides*, and *Phytophthora infestans* (Zhao et al., 2018). Similarly, DIMBOA and its derivatives are found to be effective against *Ralstonia solanacearum* and *Rhizoctonia solani*, which are the causative agents of bacterial wilt and sheath blight disease, respectively (Song et al., 2011; Guo et al., 2016). Besides, cyanogenic glucosides, such as, linamarin, lotaustralin, and dhurrin, have been shown to exhibit anti-herbivore and anti-insect properties (Gleadow and Møller, 2014). The removal of the saccharide moiety by glucosidase from cyanogenic glucosides and DIMBOA is essential for its activity. Plants sequester specific glucosidases in plastids to avoid auto toxicity. When cells get ruptured due to mechanical injury, these enzymes get released and cleave glucose moiety to induce defense responses (Gleadow and Møller, 2014). Few insects such as burnet moth can ingest cyanogenic glucosides and use it for its own defense, and also this moth has been shown to possess cyanogenic glucoside-producing genes and capable of synthesizing them on its own (Jensen et al., 2011). Cyanogenic glucoside gene machinery might have co-evolved with insects to develop an immunity against plant defense.

Metabolites, such as α-tomatine and tomatidine, display neurotoxic effects in humans by producing truncated proteins and deactivating proteasomes (da Silva et al., 2017). Unripe fruits are known to produce relatively high amounts of α-tomatine and during the process of ripening it gets gradually converted into esculeoside A, while the disruption of α-tomatine biosynthesis leads to the development of deformed fruits (Itkin et al., 2011). SGAs also possess anticancer, anti-inflammatory, antioxidant, and cardiovascular curative properties (Friedman, 2013; Al Sinani and Eltayeb, 2017). α-tomatine suppressed the metastasis of human lung cells by downregulating focal adhesion kinase (FAK), phosphatidylinositol 3-kinase (PI3K), and nuclear factor kappa B (NF-κB), which are potentially involved in cancer cell migration (Shieh et al., 2011). Interestingly, *Fusarium oxysporum-*derived tomatinases detoxify the effects of α-tomatine and suppresses host defense mechanisms (Ito et al., 2004). It can be postulated that pathogenic fungi might produce these enzymes as adaptive mechanisms to counteract host immune responses.

Casbene-derived metabolites, such as prostratin and ingenol-3,20-dibenzoate (IDB), are known to activate protein kinase C- (PKC-) mediated signaling, which is involved in repolarizing cardiac muscle cells, and could be used for treating cardiovascular disorders (Jiang et al., 2018). Prostratin and Ingenol derivatives are known to be potential latency reversal agents (LRA) and are found to clear the latently infected cells of HIV (Sloane et al., 2020). In addition, casbene-derived ingenol mebutate upsurges the neutrophil-mediated tumor cell degradation of subcutaneous melanoma (Braun et al., 2018). Noscapine, a notable BIA, is analgesic and used in the preparation of cough syrups due to its antitussive properties (Winzer et al., 2015). Thebaine is widely used in industries to develop semisynthetic drugs, such as oxycodone, oxymorphone, etorphine, nalbuphine, and naloxone, which are used as analgesics and also in the treatement of opoid poisoning (Hagel and Facchini, 2013; Singh et al., 2019). Metabolic engineering approaches for the heterologous expression of these clusters could be developed for the heterologous production of these metabolites for agriculture and pharmaceutical applications.

## Plant Gene Clusters for Metabolic Engineering

The biosynthesis of specialized metabolites in plants underpins many traits of ecological, pharmaceutical, and agronomic importance. However, plant-derived products have not gained importance till the recent times due to the domination by synthetic chemical analogs. Recent era has evidenced the emergence of plant-based natural compounds as the potential alternatives against synthetic counterparts in both agricultural and pharmaceutical sectors. Furthermore, advances in the omics approach led to the identification of plant metabolic gene clusters involved in the biosynthesis of terpenoids, alkaloids, benzoxazinoids, and cyanogenic glycosides, that have several applications. Novel findings from several studies shed light on developing sustainable metabolic engineering strategies for the overproduction of some of these specialized metabolites to meet market demands. In this regard, *in silico* approaches, including cluster predicting tools, such as PhytoClust, plantiSMASH, ClusterFinder, and ClustScan to name a few, have accelerated the discovery of diverse metabolic pathway-related gene clusters in plants (Chavali and Rhee, 2018). Technological advances in genome sequencing and availability of high-throughput functional genomics tools resulted in a shift from a single-step characterization to the validation of entire metabolic pathways. Increased knowledge of metabolic gene clusters accelerated genome engineering strategies for the biosynthesis of alkaloids and terpenoids in heterologous host systems. Nevertheless, low precursor availability, the accumulation of unwanted intermediates, and hindrance due to the lack of information regarding genes/regulatory steps are some of the bottlenecks to overproduce these compounds through synthetic biology approaches.

Primarily, the biosynthetic pathways of plant specialized metabolism are complex with several gene cascades, enzymatic reactions, and compartmentalized intermediates and or end products. Hence, a thorough understanding of the pathway dynamics is necessary to develop a strategy for heterologous metabolite production. For instance, the introduction of 12 artemisinin biosynthetic genes into tobacco chloroplasts severely affected the growth of the plant (Saxena et al., 2014). However, dividing the pathway and expressing the genes into two compartments (plastids and cytosol) successfully yielded a reasonable titer of artemisinin in tobacco that could be further used for commercial application (Malhotra et al., 2016). De La Peña and Sattely (2021) increased the biosynthesis of momilactone by expressing plastid localized geranylgeranyl diphosphate synthases (GGPPS) and CPS in cytosol through plastid tag truncation (De La Peña and Sattely, 2021). Another strategy is to modulate the precursor flux by engineering additional copies of rate limiting step genes or by silencing the branch point genes so that the flux can be diverted to the synthesis of the desired metabolite. This approach was attempted in *Artemisia annua* by downregulating the expression of β*-caryophyllene synthase* gene to increase the bioproduction of artemisinin (Lv et al., 2016). A similar strategy of pathway shunting can be achieved by gene editing tools, such as CRISPR-Cas9, which is currently being used widely in metabolic engineering (Sabzehzari et al., 2020). Furthermore, the identification of TFs that regulate multiple steps in a biosynthetic pathway is yet another approach for overproducing specific metabolites. GAME9 TF of the SGA cluster regulates both the MEP pathway and SGA pathway (Cárdenas et al., 2016). Finally, improving the cell numbers in the plant, such as increasing the trichome on the leaves, can also improve the metabolite bioproduction specific to a particular tissue (Fu et al., 2018).

The use of robust microbial hosts, such as *Escherichia coli* and yeast, is another promising approach for overproducing target metabolites by synthetic biology (Pyne et al., 2019). Celedon et al. (2016) engineered a multistep pathway in yeast cells for producing sandalwood oil similar to the one extracted from the heartwood of *Santalum album*. In addition, opoids were successfully produced in yeast through the introduction of morphine biosynthesis genes (Galanie et al., 2015). Nevertheless, some of the challenges which the researchers should anticipate and foresee while using a microbial host include: (a) ways to avoid the accumulation of toxic metabolic intermediates; (b) a fine control of gene expression; and (c) precursor limitation. Taken together, the combination of *in silico* analysis, the availability of robust functional genomic tools, and the knowledge about metabolic gene clusters could shed light into new directions in synthetic biology research at an accelerated level. The present scenario in this area of research holds a great promise to translate the basic knowledge of plant metabolism into tangible benefits for agricultural and pharmaceutical applications.

## Author Contributions

RB, SRK, and AS wrote the manuscript. RS revised it. All authors contributed to the article and approved the submitted version.

## Conflict of Interest

The authors declare that the research was conducted in the absence of any commercial or financial relationships that could be construed as a potential conflict of interest.

## Publisher's Note

All claims expressed in this article are solely those of the authors and do not necessarily represent those of their affiliated organizations, or those of the publisher, the editors and the reviewers. Any product that may be evaluated in this article, or claim that may be made by its manufacturer, is not guaranteed or endorsed by the publisher.
